# Survey of evidence-based nursing practice readiness for scoliosis-specific rehabilitation exercise in orthopedic nurses

**DOI:** 10.3389/fmed.2025.1649858

**Published:** 2025-11-28

**Authors:** Binglin Li, Ruiman Xu, Shina Cheng, Mingkui Shen, Meng Li, Huawei Li

**Affiliations:** 1 Department of Nursing, The Third People’s Hospital of Henan Province, Zhengzhou, China; 2 Yanbian University, Yanji, Jilin, China; 3 School of Nursing, Changchun Medical College, Changchun, Jilin, China

**Keywords:** adolescent idiopathic scoliosis, readiness for evidence-based nursing practices, evidence-based practice, rehabilitation nursing, clinic readiness for evidence-based nursing assessment

## Abstract

**Objective:**

The purpose of this study was to investigate the status of the readiness of evidence-based practice programs for adolescent idiopathic scoliosis, identify the influencing factors that will be faced in the implementation process, and promote the transformation of evidence.

**Methods:**

Investigate the readiness of evidence-based practice plan for adolescent scoliosis patients: clinic readiness evidence-based nursing assessment was conducted on nurses from the Department of Spine Surgery, Rehabilitation Medicine and Pediatric Department of Y City Hospital in China using the general information questionnaire and the evidence-based nursing practice readiness rating scale to evaluate the evidence-based practice readiness of the program of “special sports rehabilitation nursing for adolescent idiopathic scoliosis” to identify the possible influencing factors during the implementation process of the program.

**Result:**

In the survey assessing readiness for an evidence-based practice plan regarding scoliosis-specific rehabilitation exercises, 177 valid questionnaires were collected. The mean total score of the CREBNA scale was 135.39 ± 12.75, with subscale scores of 54.08 ± 5.34 for evidence, 39.51 ± 4.09 for organizational environment, and 41.80 ± 5.38 for facilitating factors. Individual item scores ranged from 3.45 to 4.98. Univariate analysis revealed statistically significant differences in CREBNA total scores based on participants’ understanding of evidence-based nursing practices and their perceived necessity. Multiple linear stepwise regression analysis further identified the perceived necessity of evidence-based nursing practice as the primary factor influencing readiness for implementation.

**Conclusion:**

The results of the survey on the readiness of evidence-based nursing practice showed that the current evidence-based nursing practice preparation of nurses for rehabilitation exercise for adolescents with idiopathic scoliosis was good and feasible. However, the study was limited to samples drawn from tertiary hospitals in Y City. Future multi-center and longitudinal studies are warranted to validate and extend these findings.

**Clinical implications:**

To investigate the readiness of nurses for the clinical practice of exercise rehabilitation for adolescent idiopathic scoliosis, so as to expand the evidence-based practice of adolescent rehabilitation nursing, enrich the content of special exercise rehabilitation nursing for adolescent idiopathic scoliosis in China, reduce the pain caused by surgical treatment to patients, and improve its clinical value, which is of great significance in promoting the good outcome of patients, providing reference for follow-up research, and updating relevant clinical practice guidelines in the future. Selection criteria and guiding programs are provided for reference.

## 1 Introduction

### 1.1 Research status of adolescent idiopathic scoliosis

Scoliosis refers to a three-dimensional deformity of the spinal structure, that is, the curvature of one or more segments of the cones of the spine in the sagittal plane, coronal plane and horizontal plane deviates from the midline of the body. The Scoliosis Research Society (SRS) uses a scoliosis angle (Cobb) greater than or equal to 10° as a diagnostic criterion (1). Adolescent idiopathic scoliosis (AIS) is a complex, multifactorial neuromusculoskeletal disorder that refers to scoliosis of unknown origin and is more common in adolescents over 10 years of age (2). It accounts for a considerable proportion of scoliosis, as high as 74.7% and 2–4% of adolescents in the world are affected by it (3), and AIS can be confirmed by X-ray to confirm the spinal deviation angle, that is, Cobb angle. Statistics from 2016 show that the global prevalence of AIS is 0.47∼5.2% (4), and the incidence of AIS is 0.74% in adolescents aged 5–20 years in China, and the prevalence rate is as high as 1.10% in the 14–year-old group. Among them, 80.07% of the patients with AIS had a Cobb angle of 10°∼19°, and 2.94% had a Cobb angle of ≥ 40°(5) In mainland China, a systematic review and meta-analysis of the prevalence of AIS showed that the overall incidence rate among primary and secondary school students was 1.02% (6) The overall incidence of AIS was 2.4%.

The investigators summarized the exercise rehabilitation program for adolescents with idiopathic scoliosis through evidence-based methods (7), including scientific assessment, intensive training, family training, patient education, etc., and provided a comprehensive guidance program for adolescent scoliosis patients as soon as possible in specific sports rehabilitation care. This study investigated the evidence-based practice readiness of nurses through the clinical implementation of special rehabilitation exercise programs, which provided strong support for the transformation of clinical evidence.

### 1.2 Status of research on evidence-based nursing practices

#### 1.2.1 Implications of readiness for evidence-based nursing practices

Evidence-based nursing is a science-based discipline that needs to be developed using the latest evidence. With the continuous development of the discipline, it is recognized that the introduction of new evidence into clinical practice is complex and uncertain (8), and that many barriers can lead to the failure of evidence-based practice, and the growing awareness of this fact forces researchers to comprehensively and effectively evaluate the evidence-based practice process before the evidence is applied, that is, to assess the readiness of evidence-based practice, to effectively identify the barriers to the implementation of evidence-based practice. Targeted intervention strategies have been developed to successfully advance evidence-based practice.

#### 1.2.2 Readiness for evidence-based nursing practices

Scholars commonly highlight the need to evaluate the effects of evidence-based practice in diverse ways. This requires a more flexible care environment that facilitates the universal and sustained implementation of evidence-based nursing practices. A clinical environment that engages, facilitates, and scientifically transforms can improve the efficiency of evidence implementation, eliminate ineffective interventions, and facilitate the cycle of evidence-based practice training. Improve the individual capacity of nursing workers, promote the acceptance of work change by nursing organizations, and jointly conduct evidence research and scientific implementation. The context in which evidence is adopted and sustained in nursing practice is critical to bridging the evidence-practice gap and improving outcomes for patients, clinicians, and health systems (9) Therefore, nursing practice readiness can help researchers better understand how to improve the evidence and better adapt it to the current clinical context before implementing it to facilitate patient recovery.

Currently, the rehabilitation exercise and nursing of adolescent idiopathic scoliosis patients are based on established nursing routines and diagnosis and treatment protocols of hospitals or departments. The treatment and nursing of these patients are a crucial component of the nursing work in the clinical spine surgery and rehabilitation department. Therefore, considering the overall prevalence of AIS ranges from 0.47 to 5.2% and applying the scientific concept of evidence-based nursing practice, additional research or ineffective interventions can be avoided (10, 11). There are many influencing factors of evidence-based nursing practice, so the evaluation of evidence-based practice readiness before carrying out evidence-based practice can effectively identify the influencing factors and formulate targeted countermeasures. The purpose of this study is to evaluate the readiness for evidence-based nursing practice prior to implementing an evidence-based rehabilitation exercise program for adolescents with idiopathic scoliosis (AIS). By assessing nurses’ knowledge, attitudes, organizational support, and contextual conditions in advance, the study aims to identify potential facilitators and barriers that may affect the successful implementation of evidence-based interventions. The findings will provide a reference for future clinical practice, supporting more scientific, standardized, and effective rehabilitation and nursing care for adolescents with idiopathic scoliosis.

## 2 Materials and methods

### 2.1 Study participants

In this study, we used the convenience sampling method to investigate nurses involved in AIS nursing in Yanbian Nursing Orthopedic Association, the rehabilitation department and pediatrics of a tertiary hospital.

Participants were registered nurses who were actively engaged in the nursing care of scoliosis patients, had at least 1 year of clinical experience in orthopedic or spinal nursing, and were currently working in a clinical setting with direct patient care responsibilities. Only those who voluntarily agreed to participate and provided informed consent were included. Nurses were excluded if they had been away from clinical practice for more than 3 months (e.g., due to leave or temporary reassignment), had minimal or no recent experience in scoliosis-related nursing care, were unwilling to complete the study procedures, or had incomplete or unreliable responses in the preliminary assessments.

### 2.2 Sample size

In this study, the Clinical Readiness of Evidence-Based Nursing Assessment(CREBNA) scale comprised 31 items, and the initial recommended sample size was calculated as 5–10 times the number of items (155–310 cases) to ensure adequate statistical power for scale validation and factor analysis (12). To account for potential data attrition, incomplete responses, and more robust analytical methods, the target sample size was expanded to 171–341 cases. The final sample consisted of 204 participants, which meets the minimum requirement of 155 cases and allows for basic psychometric evaluations.

### 2.3 Instruments

#### 2.3.1 General information questionnaire

This research tool consists of self-designed general data, two parts of the Evidence-based Nursing Practice Readiness Assessment Scale (Supplementary Annex 1). Part 1: General Information Table, Contents: Gender, Age, Department, Last Education, Graduate School, Years of Experience, Position, Professional Title, Degree of Understanding of Evidence-Based Nursing Practice Research experience and whether to participate in evidence-based knowledge training.

#### 2.3.2 Clinical readiness of evidence-based nursing assessment

Part II: Clinical Readiness of Evidence-Based Nursing Assessment (CREBNA Supplementary Annex 2): designed and tested by Miao et al. (13). It is the first evidence-based nursing practice readiness assessment tool in China, including three subscales, namely the evidence subscale and the organizational environment subscale, and the facilitator subscale, with a total of 31 items. The scale uses a Likert 5-level score (11). The Cronbach coefficient of the tested scale was 0.959, the Cronbach coefficient of the subscale was 0.915–0.940, and the test-retest reliability was 0.917. The content validity index was 0.976 with good construct validity.

### 2.3 Data collection methods

Firstly, the survey area and hospital were determined, and the investigators went to the relevant departments of the hospital to distribute the questionnaire by themselves and introduced the research situation and the specific requirements for filling in the questionnaire content in detail. From July 2023 to September 2023, a questionnaire was distributed to nurses in Y city who met the inclusion criteria through the work WeChat group. In the end, a total of 204 questionnaires were distributed, all of which were recovered, of which 177 valid questionnaires were recovered, with an effective recovery rate of 86.8%.

### 2.4 Statistical analysis

SPSS 26.0 software was used to descriptively count the general data and CREBNA scores of the survey subjects, in which the percentage was used for the counting data, and the mean, standard deviation (Mean ± SD) were used for the continuous data. Univariate analysis and multivariate analysis were used to analyze the influencing factors of evidence-based nursing practice readiness, and the univariate analysis method used analysis of variance, and the statistically significant variables in the univariate analysis were independent variables, and the difference was statistically significant, with *P* < 0.05 indicating that the difference was statistically significant. Multivariate linear regression was conducted to identify significant predictors of EBNP readiness after verifying regression assumptions (linearity, normal residuals, homoscedasticity, VIF < 5). Model fit was assessed using R^2^, with standardized β coefficients indicating predictor contributions, maintaining *p* < 0.05 significance (two-tailed).

### 2.5 Ethical considerations

It is important that this cross-sectional study adheres to the principles outlined in the Declaration of Helsinki, and the study protocol received approval from the ethics committee of Yanbian University (ethic No.2023–1040).

## 3 Results

### 3.1 General information

Among the 177 participants, 176 were females and 1 male, aged between 22 and 52 years, with a mean age of 32.67 ± 5.01 years; The average number of years of working experience in rehabilitation therapy is 10.65 ± 5.45 years; Among the professional titles, 89 nurses were the majority, accounting for 50.3% of the total. The proportion of respondents with a bachelor’s degree was the highest, accounting for 75.7% of the total number of respondents. 111 people had only heard of evidence-based nursing practices, accounting for 61.7% of the study participants; The vast majority of the respondents had no practical experience in scientific research, accounting for 94.4%; only a small number of respondents (about 33.9%) have participated in evidence-based knowledge training, and the vast majority of respondents believe that it is necessary to carry out evidence-based nursing practice, accounting for 75.1% (Table 1).

**TABLE 1:** General information questionnaire (*n* = 177).

Variables	*n*(%)
Age (mean, SD)	(32.67, 5.01)
Clinical experience (mean, SD)	(10.65, 5.45)
**Gender**	
Male	1 (0.6)
Female	176(99.4)
**Professional titles**	
Registered nurse	12(6.8)
Senior nurse	89(50.3)
Nurse supervisor	65(36.7)
Associate chief nurse	10(5.6)
Chief nurse	11(6.2)
**Position**	
Nurse	148 (83.6)
Nursing team leader/teaching assistant	16(9)
Matron	13(7.3)
**Educational level**	
Secondary vocational education	1(0.6)
Associate degree	8(4.5)
Bachelor’s degree	134(75.7)
Master’s degree	34(19.2)
**Scientific research experience**	
Yes	10(5.6)
No	167(94.4)
**Knowledge of evidence-based nursing practice**	
Not at all familiar	20(11.3)
Slightly familiar	111(61.7)
Moderately familiar	42(23.7)
Very familiar	4(2.3)
**Attendance of EBP lecture**	
Yes	60(33.9)
No	117(66.1)
**The need to develop evidence-based nursing practices**	
Yes	133(75.1)
No	44(24.9)

### 3.2 Evidence-based nursing practice readiness scores

The average score of the CREBNA scale was 135.39 ± 12.75, of which the average score of the evidence subscale was 54.08 ± 5.34, and the item with the highest score in this dimension was “the evidence does not violate national policies and laws and regulations” (item 8). The average overall score of the organizational environment subscale was 39.51 ± 4.09, and the item with the highest score in this dimension was the item “I have good execution ability for tasks assigned by superiors” (item 19). The average overall score of the facilitator subscale was 41.80 ± 5.38, and the item with the highest score in this dimension was “facilitators in the evidence-based practice team who were able to develop practical evidence-based practice programs” (item 23). See Table 2 for the specific scores and rankings.

**TABLE 2:** CREBNA scores (*n* = 177).

No.	Items	Mean	SD	Ranks
	**Evidence subscale (12 items)**	54.08	5.34	
1	The sources of evidence are reliable	4.51	0.59	6
2	The evidence was assessed through a rigorous quality assessment process	4.60	0.56	3
3	The evidence is appropriate for patients in institutions where evidence-based practice protocols are about to be implemented	4.61	0.58	2
4	The selection of evidence was based on a combination of work experience and professional judgment of clinical nursing staff	4.53	0.61	5
5	The evidence was screened taking into account the needs of patients	4.57	0.57	4
6	The use of this evidence can lead to recovery and direct or indirect improvements in patient outcomes	4.50	0.57	9
7	The implementation of this evidence can improve the quality of care/care services	4.51	0.57	7
8	The evidence does not violate national policies and laws and regulations	4.62	0.59	1
9	The evidence was screened taking into account current medical conditions and the level of care	4.50	0.60	8
10	The evidence addresses the question of the scope of medical/nursing responsibilities and the ability to intervene in a way that can be done accordingly	4.45	0.58	11
11	I am happy to accept the application of this evidence to the clinic, which is in line with my own requirements and values	4.50	0.53	10
12	Evidence has been transformed into forms that are easy to disseminate and easy to understand and apply, such as processes, practice manuals, etc.	4.18	0.73	25
	**Organizational environment subscale (9 items)**	39.51	4.09	
13	Leaders are adept at actively exploring and improving clinical work	4.30	,0.62	21
14	The leader has a good influence and we are willing to carry out her/his suggestions or orders	4.38	0.60	19
15	Leaders are able to allocate human resources appropriately based on clinical work	4.39	0.59	17
16	Leaders have good communication and coordination skills	4.42	0.57	13
17	Leaders are able to listen to our opinions and perspectives broadly	4.38	0.61	18
18	I am willing to experiment with new clinical care processes, methods, techniques, etc.	4.40	0.55	16
19	I have a good ability to execute the tasks assigned by my superiors	4.44	0.53	12
20	Our team members are able to work together to achieve specific goals	4.41	0.53	15
21	My ward has a multidisciplinary culture and workflow	4.41	0.57	14
	**Facilitator subscale (10 items)**	41.80	5.38	
22	Facilitator with extensive expertise and clinical experience in the evidence-based practice team	4.29	0.62	22
23	The evidence-based practice team includes facilitators who can develop practical evidence-based practice protocols	4.31	0.64	20
24	The forthcoming evidence-based practice has been integrated into all stakeholders (e.g., investigators, doctors, nurses, and other members of a multidisciplinary team)	4.16	0.66	27
25	Incentives (e.g., job prospects, learning opportunities, collective honors, remuneration, etc.)	4.02	0.78	31
26	There are various forms associated with evidence-based practice projects (e.g., lectures, video lectures, seminars, hands-on exercises).	4.07	0.74	30
27	I had the opportunity to get involved in the ward (make/change workflows, resource allocation, staffing, etc.).	4.16	0.70	26
28	Soon/completed evidence-based practice projects are supported by a superior leadership (hospital/nursing).	4.12	0.70	28
29	My ward has the information resources needed to conduct evidence-based practice (medical data, technician support, etc.)	4.10	0.66	29
30	We have a feedback system to optimize the practice plan based on feedback from clinical nursing staff and patients	4.24	0.62	23
31	Have plans to promote evidence-based practice	4.23	0.63	24
Total volume		135.39	12.75	

### 3.3 Univariate analysis of evidence-based nursing practice readiness

There was no significant difference in the number of years of work experience among nurses in the comparison of CREBNA total scores (*P* = 0.128). There was no significant difference in the total scores of different job titles (Registered Nurse/Senior Nurse/Nurse Supervisor/Associate Chief Nurse/Chief Nurse) (*P* = 0.155). There was no significant difference in the total score of different academic qualifications (Secondary Vocational Education/Associate Degree/Bachelor’s Degree/Master’s Degree) (*P* = 0.103). There were statistically significant differences in the CREBNA total score for different job positions (Nurses/Nursing team leaders, Teaching assistants/Matron), whether they had scientific research practice experience, different levels of understanding of evidence-based nursing practices, and whether they had participated in evidence-based knowledge training (*P* < 0.05) (Table 3).

**TABLE 3:** Univariate analysis of influencing factors of CREBNA (*n* = 177).

Item	Scores		F/t	*P*
	Mean	SD		
**Professional titles**			1.27	0.155
Registered nurse	130.17	17.10		
Senior nurse	135.42	11.92		
Nurse supervisor	136.80	12.13		
Associate chief nurse	133.50	11.60		
Chief nurse	123.0	0		
**Position**			1.563	0.029*
Nurse	135.99	12.58		
Nursing team leader/teaching assistant	133.94	10.18		
Matron	130.38	16.80	1.348	0.103
**Educational level**				
Secondary vocational education	129.00	0		
Associate degree	132.25	22.29		
Bachelor’s degree	134.74	12.80		
Master’s degree	138.88	9.11		
**Scientific research experience**			1.081	0.361
Yes	136.70	21.53		
No	135.31	12.75		
**Knowledge of evidence-based nursing practice**			5.558	0.001*
Not at all familiar	128.40	17.34		
Slightly familiar	134.36	11.86		
Moderately familiar	141.10	10.35		
Very familiar	139.00	13.10		
**Attendance of EBP lecture**			1.608	0.022*
Yes	140.12	10.16		
No	132.97	13.29		
**The need to develop evidence-based nursing practices**			1.787	0.007*
Yes	137.33	12.30		
No	129.52	13.40		

SD, standard deviation.

*p < 0.05 (two-tailed).

### 3.4 Multiple linear stepwise regression analysis of the influencing factors of evidence-based nursing practice readiness

#### 3.4.1 Explanation of variable assignment of factors influencing readiness in evidence-based nursing practice

In order to exclude confounding factors, the total score of nursing evidence-based practice readiness was used as the dependent variable (Y), and four statistically significant factors in the univariate analysis (position, degree of knowledge of nursing readiness practice, participation in evidence-based knowledge training, and necessity to carry out evidence-based nursing practice) were taken as independent variables (X Multiple linear regression analysis was carried out, and the independent variables were entered by stepwise regression analysis, and the variables were transformed according to the requirements of the regression model for the variables, and the assignment of each variable is shown in Table 4.

**TABLE 4:** Influencing factors and assignment of evidence-based nursing practice preparation.

Factors	Variable name	Assignment method
Attendance of EBP lecture	X_1_	No = 1; yes = 2
Knowledge of evidence-based nursing practice	X_2_	Not at all familiar = 1; Slightly familiar = 2; Moderately familiar = 3; Very familiar = 4
The need to develop evidence-based nursing practices	X_3_	No = 1; Yes = 2
Position	X_4_	Set the subvarieties with “nurse” as the reference Leader/Teaching Assistant (X_41_) X_41_ = 1; X_42_ = 0 matron (X_42_) X_41_ = 0; X_42_ = 1

#### 3.4.2 Multiple linear stepwise regression analysis of the influencing factors of readiness in evidence-based nursing practice

The results of multiple linear stepwise regression analysis showed that the degree of understanding of evidence-based nursing practice (X_2_) and the necessity of evidence-based nursing practice (X_3_) were important factors affecting the total score of CREBNA (P < 0.05). Among them, the necessity of carrying out evidence-based nursing practice (X_3_) had the greatest impact on the total score of CREBNA, and the degree of understanding of evidence-based nursing practice (X_2_) and the understanding of evidence-based nursing practice process had less impact on it. The regression equation Y = 123.24 + 4.828X_2_ + 6.571X_3_, which could explain 11.8% of the variation (R^2^ = 0.118). That is, the readiness of evidence-based nursing practice is positively correlated with the degree of understanding of evidence-based nursing practice and the necessity of carrying out evidence-based nursing practice (Table 5).

**TABLE 5:** Stepwise multiple linear regression analysis of factors affecting CREBNA (*n* = 177).

Item	B(SE)	Beta	t	95% CI for B	*P*-value
Constant	123.24 (4.48)		25.289	114.42–132.06	< 0.001
Knowledge of evidence-based nursing practice (X_2_)	4.828 (2.11)	0.223	3.109	2.67–6.99	0.001*
The need to develop evidence-based nursing practices (X_3_)	6.571 (1.42)	0.244	3.400	4.77–8.37	0.002*

Model statistics: *R*^2^ = 0.118, *P* < 0.001. B, unstandardized coefficient; E, standard error; β, standardized coefficient; CI, confidence interval. All significant predictors (*P* < 0.01) were retained through stepwise selection (entry *P* < 0.05, removal *P* > 0.10). The model explains 19% of variance in readiness scores.

*P < 0.05 indicates a statistically significant difference.

## 4 Discussion

### 4.1 Analysis of the current situation of nurses’ general data

A total of 177 nurses and pediatric nurses in the rehabilitation department of Y city were included in this study, and the proportion of females was much higher than that of males (14), including 176 females (99.4%) and 1 male, accounting for 0.6%, which was consistent with the results of Zhang Romem’s study (15) and Lindsay’ study (16) In terms of professional titles, the proportion of senior and deputy senior is small, 0.6 and 5.6% respectively, which is consistent with the results of Liu’s (17) 2021 survey on China’s nursing human resources, nurses and nurses in charge account for a relatively large proportion, accounting for 87%, which is higher than the national level, but it has not changed the world *status quo* of the disparity in the structure of nurses’ medical titles; 94.9% of the respondents have a bachelor’s degree or above, which is much higher than the national level, indicating that nursing staff have great advantages in their ability to deal with difficult and complex problems and nursing scientific research and innovation ability, and the level of nursing discipline is relatively high. Most of the respondents have no scientific research experience, which may be related to the late start of the nurse profession in China and the large room for development (18); 61.7% of nurses have heard of evidence-based nursing practice, and 33.9% of nurses have participated in evidence-based knowledge training, which is related to the level of education (19). 75.1% of the nurses believed that it was necessary to carry out evidence-based nursing practice, which was related to the gradual development of nursing research in China on the right track, separated from the secondary disciplines, and rapidly developed (20).

### 4.2 Survey of the current state of nurses’ nursing practice readiness

In the field of nursing evidence-based, the application of evidence is the bridge between practice and theory, linking theory and practice, and conducting a scientific, comprehensive and objective evaluation before the application of the best evidence can help researchers predictively identify the obstacles and difficulties in the application of evidence, so as to promote the transformation of evidence. In this study, the CREBNA scale was used to conduct a comprehensive evaluation of three subscales: evidence, organizational environment, and facilitating factors. The high total score on the CREBNA scale (135.39 ± 12.75, or 87.35% of the total possible score) reflects a strong readiness for implementing the scoliosis rehabilitation practice program in several ways. According to Huang Miao’s study (13), the total CREBNA score was 136.93 ± 13.59, accounting for 87.7% of the full score, which suggests a high level of readiness for implementing the research plan. Similarly, in the study conducted by Song (21), the total score reached 141.51 ± 5.33, representing 91.30% of the full score, further indicating a strong preparation level for executing the proposed intervention. It shows that the practice program of scoliosis special sports rehabilitation nursing has a relatively high degree of preparation, and the current environment is conducive to the development of evidence-based practice programs. With the development of evidence-based practice in China, more clinical nurses have begun to realize the importance of evidence-based nursing in guiding clinical practice and are therefore more willing to participate in evidence-based nursing practice actively. This trend not only reflects the professional progress of the nursing industry but also reflects the continuous pursuit of clinicians to improve the quality and effectiveness of patient care.

### 4.3 Analysis of differences in nurses’ nursing practice readiness in the general data

#### 4.3.1 The position of the research subject

In the univariate analysis of this study, there were statistically significant differences in the scores of nurses in terms of positions (department nurse, nursing team leader/teaching assistant, nurse manager). It can be concluded that the position of nurses is an influential factor in their nursing practice readiness, which is consistent with the research results of Konlan et al. (22). The reason for this may be that the job responsibilities and nursing tasks undertaken by nurses in different positions may be different in the first place. Nurses with the position of nursing team leader/teaching assistant had higher scores of evidence-based nursing practice readiness than ordinary nurses, considering that they had more teaching experience and clinical management experience than department nurses compared with nursing team leaders/teaching assistants, could take the initiative to learn in teaching and clinical management activities, had more contact with patients’ daily care, and were easy to find nursing problems. The department nurse is usually responsible for the day-to-day nursing work of a specific department, the nursing team leader or teaching assistant may be responsible for more team management and education training tasks, and the nurse manager is responsible for managing and directing the entire nursing team (23). Secondly, nurses in different positions face different job pressures and risks. For example, the nurse manager, as the leader of the team, needs to take on greater decision-making responsibility and pressure, while also dealing with coordination and management issues within the team. In addition, nurses in different positions also differ in their remuneration packages, promotion opportunities, etc., which may lead to differences in their motivation and engagement in their work, which can further improve the evidence-based nursing practice readiness score. This study argues that nurse managers are mainly engaged in administrative work in the department, and their role as clinical nursing leaders is crucial, and their influence on the readiness of clinical nursing practice cannot be ignored, so as to improve their evidence-based nursing capabilities, overcome obstacles in the process of evidence-based practice, establish an evidence-based nursing practice culture in clinical work, and effectively lead evidence-based nursing.

#### 4.3.2 The extent to which participants understood evidence-based nursing practices

In the univariate analysis of this study, there was a statistically significant difference in the scores of nurses in terms of their level of knowledge of evidence-based nursing practices. This finding reveals significant differences in the perception of evidence-based nursing among the nurse population. First, nurses have different educational backgrounds, work experience, and expertise, which can lead to gaps in their understanding and perception of evidence-based nursing (24, 25). Nurses with higher education or professional training may be more receptive to and understand the concepts and methods of evidence-based nursing, while some nurses with rich experience but lack systematic learning may have relatively little knowledge of evidence-based nursing. Second, the degree to which evidence-based care is promoted and applied may vary from hospital to hospital or department. Some hospitals may pay more attention to the practice and research of evidence-based nursing, providing nurses with more learning and practice opportunities, thereby improving their understanding of evidence-based nursing; Some hospitals may not be able to effectively promote evidence-based care for a variety of reasons, resulting in a lack of understanding among nurses. In addition, factors such as individual nurses’ attitudes toward learning, interests, and job demands may also affect their understanding of evidence-based nursing. Some nurses may be more focused on clinical practice and patient needs, keeping an open and learning attitude toward new nursing concepts and approaches; Some nurses may lack interest or motivation to learn about new nursing concepts due to their busy schedules or other reasons, and the impact of this difference in understanding on nursing practice is significant. Nurses with a better understanding of evidence-based nursing are more likely to apply this concept in practice to improve the quality of care and patient satisfaction. Nurses with less knowledge may still rely on traditional methods of care and are unable to make the most of the available scientific evidence to guide practice. In conclusion, the more they understand the evidence-based nursing practice, the better the level of preparedness of evidence-based nursing practice, and it is suggested that nursing managers in various departments should create a positive evidence-based nursing practice environment in the department, provide evidence-based nursing database resources, and promote the improvement of the nursing evidence-based practice readiness of rehabilitation and pediatric nurses (26). Research shows that (27) organizational culture and readiness for evidence-based practices play a crucial role in driving evidence-based practice. As a branch of evidence-based practice, evidence-based nursing started late in China and developed relatively slowly, so it needs to be transformed at the policy and practice levels to support evidence change.

#### 4.3.3 Whether respondents had participated in evidence-based training

In the one-way analysis of this study, there was a statistically significant difference in the scores of nurses in whether they participated in evidence-based knowledge training. It can be concluded that whether nurses have participated in evidence-based knowledge training is the influencing factor of their nursing practice readiness. Nurses who participated in evidence-based knowledge training had higher scores of nurses’ practice readiness than those who did not participate in evidence-based knowledge training, so it was recommended to strengthen the education and training of evidence-based nursing and improve the professional quality and cognitive level of nurses. Promote the practice cases and successful experiences of evidence-based nursing and stimulate the interest and motivation of nurses in learning (28); Establish a practice guidance and evaluation system for evidence-based nursing to provide specific guidance and support to nurses. Through these measures, we can gradually narrow the gap in nurses’ understanding of evidence-based nursing, promote the continuous improvement and innovative development of nursing practice, promote the improvement of nursing staff’s evidence-based nursing ability in clinical evidence-based nursing practice (29), and guide nurses to combine evidence-based concepts and methods in the process of clinical nursing to carry out evidence-based nursing (30).

#### 4.3.4 Whether participants felt the need for evidence-based nursing practice

In the univariate analysis of this study, there was a statistically significant difference in the scores of nurses who considered it necessary to carry out evidence-based nursing practice. It can be concluded that the necessity of evidence-based nursing practice is the influencing factor of nursing practice readiness.

First of all, there is the difference in educational background and professional training. Nurses who are educated in systematic evidence-based nursing are more likely to recognize its importance and have a positive attitude. Nurses who lack the education and training may have insufficient understanding or skepticism about its necessity (31). Secondly, differences in clinical experience can also affect nurses’ cognition. Nurses with extensive clinical experience are more likely to recognize the important role of evidence-based nursing in improving the quality of care, reducing errors and unnecessary interventions. In contrast, less experienced nurses may not have yet to appreciate these advantages (32). In addition, the work environment and hospital culture are also key factors influencing nurses’ attitudes. In a healthcare environment that values scientific research and encourages innovation in practice, nurses are more likely to embrace and identify with the concept of evidence-based nursing (33). Conversely, if the hospital has a conservative culture and lacks acceptance of new ideas and methods, nurses may have reservations or objections to it. Finally, nurses’ personal values and career pursuits can also influence their perceptions of the necessity of evidence-based nursing. Nurses who pursue professional advancement and focus on the interests of their patients are more likely to see evidence-based nursing practices as necessary. The significance of this difference in scores is that it reveals the different cognitive and practical needs of the nurse population. For managers and educators, individualized training and mentoring strategies are needed to address these differences to increase nurses’ awareness and acceptance of evidence-based nursing practices. At the same time, it is also necessary to promote mutual learning and progress among nurses through the sharing of practical cases and experience exchange. Therefore, it is recommended to carry out training on evidence-based knowledge to improve nurses’ evidence-based ability, deepen nurses’ understanding of evidence-based practice, and then choose to carry out evidence-based nursing practice.

### 4.4 Analysis of influencing factors of nursing practice readiness of study subjects

In the univariate analysis of this study, there were statistically significant differences in the CREBNA score of whether they had participated in evidence-based knowledge training and the position of nursing staff, but the variables did not enter the regression equation, indicating that the training and position of evidence-based knowledge were not independent influencing factors of CREBNA, and may also be related to the small sample size of the leaders of this study, and the specific reasons need to be further explored. The results of stepwise regression analysis showed that the necessity of evidence-based nursing practice was the main factor affecting the readiness of evidence-based practice, which was consistent with the results of Yoo et al.’s study (34). The reason for this analysis is that nurses’ concept and understanding of evidence-based nursing are of great significance to the innovation of nursing clinical work, and evidence-based nursing can guide clinical work, reduce nursing burden, and save related costs. The review by Squires et al. (35) showed that the strength of belief and attitudes of implementers were important factors influencing evidence transfer. It is suggested that nursing managers should create a positive evidence-based nursing environment, shape their own evidence-based nursing culture, carry out multi-form publicity activities of evidence-based nursing concepts, and adopt a point-to-point approach to let people with positive attitudes make successful cases, so as to gradually promote the transformation of other nurses from “empirical nursing” to “evidence-based nursing.”

## 5 Implications for educational practice and policy

This study highlights the importance of enhancing nurses’ readiness for EBP through education, training, and policy support. Nursing programs should integrate EBP concepts, critical appraisal skills, and clinical application strategies across undergraduate and continuing education. Healthcare institutions should provide structured training, accessible evidence resources, and mentoring, while encouraging nurse managers to model EBP and foster a supportive departmental culture. At the policy level, administrators should ensure protected time, incentives, and performance evaluation linked to EBP implementation. These measures can strengthen nurses’ EBP competence, improve the quality of adolescent scoliosis rehabilitation, and promote sustainable evidence-based nursing practice.

## 6 Limitations

This study has several limitations that should be acknowledged. First, the sample was exclusively drawn from tertiary hospitals in Y City, which may limit generalizability to primary or secondary healthcare settings, potentially overestimating EBP readiness in less-resourced institutions. Second, socioeconomic factors—including regional economic disparities, hospital funding, and patient socioeconomic status—were not accounted for, though they may significantly influence EBP implementation. Third, while the CREBNA scale assessed organizational environment, deeper institutional factors such as leadership investment in nursing innovation and workplace culture variations were not fully explored. Additionally, the findings reflect China’s unique healthcare context and require cross-cultural validation before broader application. Finally, while key influencing factors were identified, future research should incorporate more granular analyses of barriers and facilitators in AIS rehabilitation management. These limitations highlight the need for multi-center studies, economic and cultural contextual analyses, and longitudinal assessments of EBP implementation.

## 7 Conclusion

In this study, we validated the readiness of orthopedic nurses for evidence-based practice in adolescent idiopathic scoliosis special sports rehabilitation nursing from three levels: environment, facilitating factors and evidence. The high total CREBNA score reflects that the scoliosis rehabilitation practice is well-prepared, supported by a favorable environment and facilitating factors, and ready to implement evidence-based interventions. This readiness is a critical foundation for the successful adoption and sustainability of the rehabilitation program. Consequently, the readiness indicated by the CREBNA scale implies that the healthcare team possesses the necessary capacity, motivation, and structural support to implement evidence-based scoliosis rehabilitation programs. This alignment is critical for optimizing clinical outcomes, including spinal stabilization, prevention of curve progression, and improvement in patients’ quality of life.

## Data Availability

The original contributions presented in the study are included in the article/Supplementary material, further inquiries can be directed to the corresponding authors.
