# Supplementary material for: Survey of evidence-based nursing practice readiness for scoliosis-specific rehabilitation exercise in orthopedic nurses
Source: Front Med (Lausanne). 2025 Nov 28;12:1649858. doi: 10.3389/fmed.2025.1649858 (PMC12698479; doi:10.3389/fmed.2025.1649858)
Supplement: Supplementary file 1 [file Table_1.docx]

--------------------------------------------------------
Comment from: Li Binglin

Q6:Not Applicable
.
Q7:Annex
Q21：7：Xiao Hong, Li Binglin, Chang Hongjuan, Huang Jing. Network meta-analysis of the interventional effects of different rehabilitation exercise trainings on patients with adolescent idiopathic scoliosis. Chinese Journal of Modern Nursing. 2025;31(4):470-477. DOI: 10.3760/cma.j.cn115682-20240131-00638:
13：Huang Miao, Gu Ying, Zhang Yuxia, Hu Yan, & Zhou Yingfeng. (2017). Development and validation of the Evidence-Based Nursing Practice Readiness Assessment Scale.
Chinese Journal of Evidence-Based Pediatrics
,
12
(2), 121-125.

--------------------------------------------------------
Comment from: Li Binglin

Q6:Not Applicable
.
Q7:For the Annex, please see the author correction.​
Q21：7：Xiao Hong, Li Binglin, Chang Hongjuan, Huang Jing. Network meta-analysis of the interventional effects of different rehabilitation exercise trainings on patients with adolescent idiopathic scoliosis. Chinese Journal of Modern Nursing. 2025;31(4):470-477. DOI: 10.3760/cma.j.cn115682-20240131-00638:
13：Huang Miao, Gu Ying, Zhang Yuxia, Hu Yan, & Zhou Yingfeng. (2017). Development and validation of the Evidence-Based Nursing Practice Readiness Assessment Scale.
Chinese Journal of Evidence-Based Pediatrics
,
12
(2), 121-125.

--------------------------------------------------------
Comment from: Li Binglin

Q6:Not Applicable
.
Q10:For the Annex, please see the author correction.​
Q21：7：Xiao Hong, Li Binglin, Chang Hongjuan, Huang Jing. Network meta-analysis of the interventional effects of different rehabilitation exercise trainings on patients with adolescent idiopathic scoliosis. Chinese Journal of Modern Nursing. 2025;31(4):470-477. DOI: 10.3760/cma.j.cn115682-20240131-00638:
13：Huang Miao, Gu Ying, Zhang Yuxia, Hu Yan, & Zhou Yingfeng. (2017). Development and validation of the Evidence-Based Nursing Practice Readiness Assessment Scale.
Chinese Journal of Evidence-Based Pediatrics
,
12
(2), 121-125.
